# Gene regulatory network analysis supports inflammation as a key neurodegeneration process in prion disease

**DOI:** 10.1186/1752-0509-6-132

**Published:** 2012-10-15

**Authors:** Isaac Crespo, Kirsten Roomp, Wiktor Jurkowski, Hiroaki Kitano, Antonio del Sol

**Affiliations:** 1 Luxembourg Center for Systems Biomedicine (LCSB), University of Luxembourg, Campus Belval, 7, avenue des Hauts fourneaux, Luxembourg L-4362, Luxembourg; 2 The Systems Biology Institute, Tokyo, 108-0071, Japan

**Keywords:** Prion disease, Inflammation, Neurodegeneration, Gene regulatory network, Perturbation, Stable states

## Abstract

**Background:**

The activation of immune cells in the brain is believed to be one of the earliest events in prion disease development, where misfolded PrionSc protein deposits are thought to act as irritants leading to a series of events that culminate in neuronal cell dysfunction and death. The role of these events in prion disease though is still a matter of debate. To elucidate the mechanisms leading from abnormal protein deposition to neuronal injury, we have performed a detailed network analysis of genes differentially expressed in several mouse prion models.

**Results:**

We found a master regulatory core of genes related to immune response controlling other genes involved in prion protein replication and accumulation, and neuronal cell death. This regulatory core determines the existence of two stable states that are consistent with the transcriptome analysis comparing prion infected versus uninfected mouse brain. An *in silico* perturbation analysis demonstrates that core genes are individually capable of triggering the transition and that the network remains locked once the diseased state is reached.

**Conclusions:**

We hypothesize that this locking may be the cause of the sustained immune response observed in prion disease. Our analysis supports the hypothesis that sustained brain inflammation is the main pathogenic process leading to neuronal dysfunction and loss, which, in turn, leads to clinical symptoms in prion disease.

## Background

Prion proteins are responsible for a class of fatal neurodegenerative diseases, which affect both humans and animals. Prion disease, like other chronic neurodegenerative disease such as Alzheimer’s or Parkinson’s diseases, belongs to the class of protein misfolding disease that are characterized, pathologically, by abnormal protein deposition and the formation of amyloid plaques [1]. Prion protein exists in major two isoforms: normal, cellular prion protein (Prion^C^) and abnormal, misfolded prion protein (Prion^Sc^). In most forms of prion disease, the misfolded isoform accumulates in extracellular aggregates. Prion disease is transmissible, the primary route of infection being through the ingestion of abnormal prions.

Several hypotheses have been put forward to explain prion disease pathogenesis, such as Prion^C^ loss-of-function, Prion^Sc^ gain-of-toxic function, endoplasmatic reticulum stress, activation of autophagy and/or apoptotic death pathways, and chronic brain inflammation induced by misfolded protein and neuronal injury [2], but none has emerged so far as the main initiator and/or propagator of the disease [3]. We have used a computational network analysis based on known gene expression data to address this complex question. Our analysis shows that it is brain inflammation that plays a key role in prion disease. The main cellular mediators of brain inflammation are microglia, which are responsible for the first active immune response in the brain. These cells are among the earliest responders but their role in prion disease initiation and progression is still debated. We have examined gene expression data from a recent comprehensive transcriptome study on the initiation and progression of prion disease in mouse [4] using a network analysis-based approach and identified a limited number of immune response- related genes as crucial factors in the disease process. These genes appear to be capable of irreversibly locking a large network of differentially expressed genes (DEG) into a disease state, thus uncovering an essential process of the early steps in disease progression.

There is increasing evidence that the brain and immune system are connected: in both normal and pathological conditions neurons are interacting with immune cells and regulating their activity [5]. Chronic immune activation, especially of microglia, is a common feature of chronic neurodegenerative conditions such as Alzheimer’s and Parkinson’s [6]. For example, there are many similarities between Alzheimer’s disease and prion disease: both diseases are characterized by the protein deposition, significant neuronal degeneration and the morphological activation of microglia and astrocytes [7]. In prion disease, neuropathological data shows that microglia are among the earliest responders to neurodegeneration [8,9] and that microglia proliferate in response to disease-causing prion protein deposition [10].

It has been proposed that diseased states correspond to abnormal stable states in the gene expression landscape, or in other words, disease is reflected by long term differential expression patterns [10][11]. The natural robustness of biological networks allows them to maintain the organism in a healthy state despite the influence of a range of external and internal perturbations. The network robustness is a topological property i.e. is a result of specific connectivity between genes in question. However, abnormal network states occasionally occur under the influence of strong internal or external perturbations (i.e. disease initiators or irritants), and these may play an important role in disease initiation and progression. Thus a particular connectivity pattern that is responsible for the robustness of the healthy state can also produce a robust diseased state.

Here we address the question of how a subset of genes forming a master regulatory core in a gene regulatory network is able to determine the stability of this network in a prion disease context. A previous study has shown that gene interactions forming small bi-stable circuits are implicated in the resilience and progression of human cancers, where the healthy and cancer states were considered to be the two stable states [12]. However, how these isolated small bi-stable circuits contribute to the general mechanism of the network stability (and hence cancer development) is still open. We address this issue by significantly extending the idea of bi-stable circuits to a more comprehensive mechanism, the so called master regulatory core, which could explain how the network shifts from the healthy to the diseased stable state. During our analysis we realized that genes belonging to the master regulatory core are highly connected in the network and largely related to immune response, supporting the idea of the central role of a sustained inflammatory process leading to neuronal dysfunction and death in the prion disease progression.

## Results

### The global and core regulatory networks

**Figure 1:**
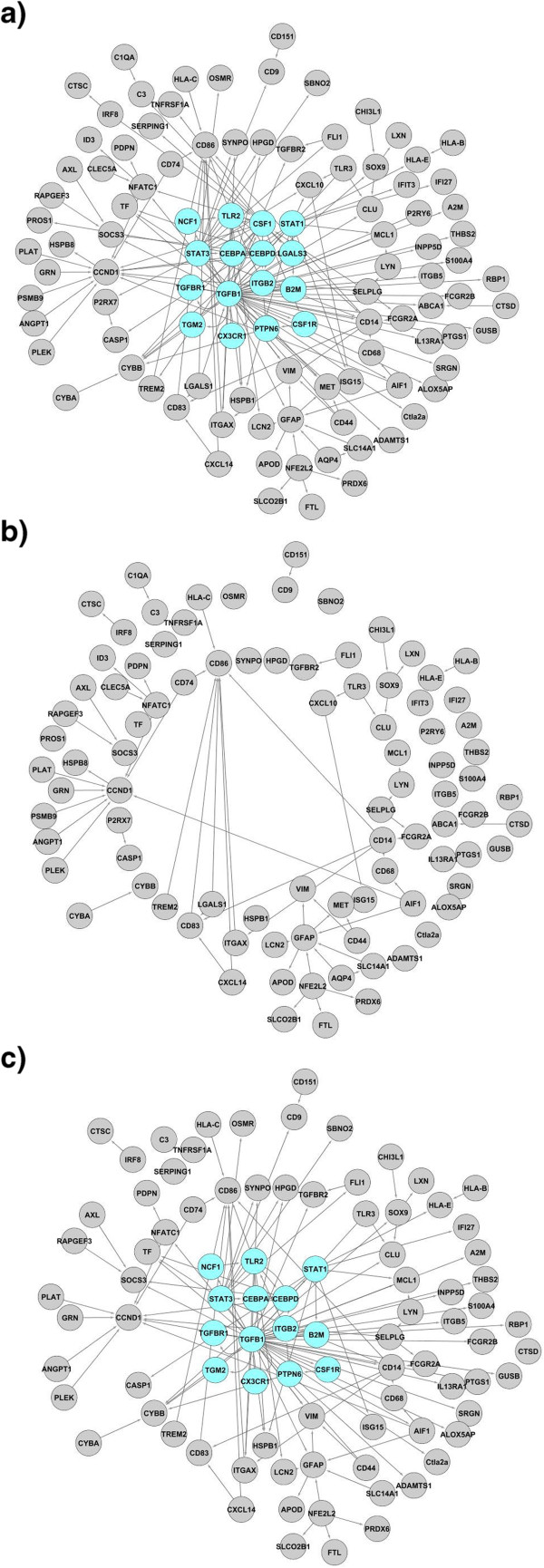
**Fragmentation analysis of the global network.** The original global unfragmented network (**a**), the impact on the network connectivity due to the removal of the sixteen genes belonging to the SCC (**b**), and an example of the removal of sixteen genes randomly selected (**c**). In (**b**) most of the genes become disconnected and the size of the giant component or the biggest connected graph is only 38 genes. In (**c**) when removing 16 randomly selected genes, the mean of the giant component was 81.02 nodes (standard deviation of 8.29) for 1000 removals . This figure illustrates the relevant role of the SCC as a connectivity element of the network.

Our initial goal was to build a gene regulatory network based on the differentially expressed genes reported by Hwang *et al.*[4]. The functional relationships, based on gene expression, found in the literature resulted in a global network consisting of 106 genes that are differentially expressed during prion infection (all upregulated), connected with 169 functional relations (all activations). (Figure 1A and Additional file 1).

We then carried out stability analysis performed using a boolean dynamical model to compute network stable states. Afterwards, we identified a set of genes able to trigger the transition between attractors and at the same time lead to the network’s persistence in the diseased state. Due to the possibility of there being incomplete information about gene-gene interactions even in the parts of the network which are well known, we based our analysis and conclusions on the network stability and the transition between stable states, avoiding a detailed description of transient states (potentially feasible given that experimental data has several time points) that are more sensitive to the lack of information.

Network dynamics are regulated by the structure of the network through the flow of information through feed-forward and feed-back loops. When we looked for network structures with a exchange of information, we found a unique strongly connected component (SCC) consisting of 16 genes. The hallmark of such a structure is that thanks to specific connectivity the information can flow from one gene to any other in the structure following at least one path (see Methods for detailed explanations). This mutual influence between any pair of genes belonging to the SCC makes this structure relevant in terms of information exchange, and therefore potentially determinant for the network’s stability. The SCC is mainly regulatory in nature with only 6 incoming functional relations. This SCC constitutes the regulatory core, and its regulatory impact extends up to 74 genes*,* so the states of these 74 genes depend on the state of the master regulatory core.

In order to analyze the stability of regulatory core genes alone, we carried out a simulation of network dynamics to determine the stable states of this sub-network in isolation using a Boolean dynamical model. Two stable states were found for the regulatory core, one with all nodes “off” and one with all nodes “on”. Extending the simulation to cover genes regulated by the regulatory core (i.e. the core network) produced consistent results: again, we found two stable states, one with all nodes “off” and one with all nodes “on”.

**Figure 2:**
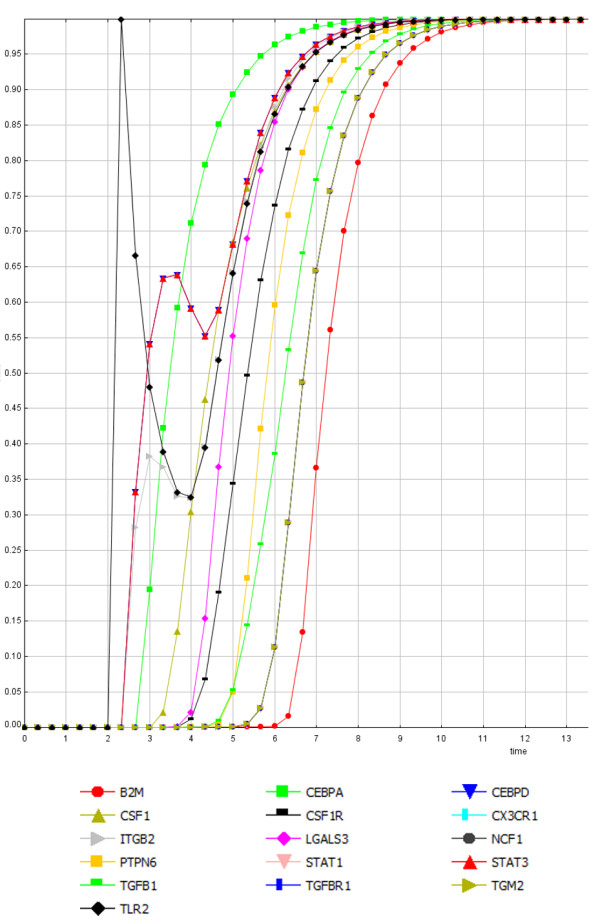
**Perturbation analysis of a gene in the SCC Perturbation of the TLR2 gene (black diamond), and its effect on the other genes of the SCC.** Y-axis: 0 indicates the “off” state, 1 indicates the “on” state. TLR2 is capable of triggering the transition from the “off” (healthy) to the “on” (disease) stable state for all genes in the SCC. The simulations were performed assuming a continuous dynamical system where the initial states are the attractors previously computed in a discrete model (Boolean). The Y-axis represents the “level of activity” in a range between 0 and 1, and X-axis represents “time” in arbitrary units.

The perturbation analysis carried out using a continuous dynamical model showed that all regulatory core genes were capable of triggering the transition from the “off” to the “on” stable states in the core network (Figure 2). But no gene was individually capable of inducing the opposite transition, from the “off” to the “on” state. Therefore, when the “on” state was reached, the system staid locked despite external influences. Only simultaneous down regulation of a set of nodes (theoretically possible but unlikely to occur in practice) affecting several circuits in the regulatory core would be able to reverse the “on” state; otherwise, the system is irreversibly activated supporting the idea that the regulatory core constitutes a master regulatory switch that can be activated by external inputs and is able to maintain the activation of a set of nodes that may be relevant for the progression of prion disease.

### Network properties

A network is constituted by nodes (i.e. genes) that are inter-connected by edges (i.e. directed functional relations); expression of some genes can either activate or inhibit expression of other genes in the network. Therefore it is important to recognize genes that have more control over the network. We applied two measures: network fragmentation and betweenness centrality to identify genes that play the role of so called communication hubs (mediators of interactions between other, more peripheral genes). Fragmentation is a measure to assess overall network connectivity and may be helpful to determine the impact of a sub-network on global topology. The fragmentation analysis of the global network produced the following results. The mean of the giant component size for 1000 randomized removals of 16 nodes was 81.02 nodes (standard deviation 8.29), while it was only 38.00 nodes in the case of SCC node removal. The difference between these values is 5.18 times the standard deviation of the random removal values. This indicates that the size of the biggest set of connected nodes was reduced dramatically when we removed the nodes of the SCC instead of a random selection of 16 nodes (Figure 1B and C). These results underlined the relevant role of the SCC as a connectivity element of the global network.

The network presented here was scarcely interconnected, which was also reflected in the betweenness centrality analysis. There was a small group of central genes (mostly belonging to SCC) that has a much larger number of peripheral genes in the network connected to them. Six genes could be considered highly central (normalized betweenness > 1): *TGFB1*, *CSF1*, *TLR2, CEBPA, LGALS3 and STAT3.* In total, 25 genes were not peripheral (i.e. they mediated at least one gene connection). There was a significant difference when the betweenness centrality of genes participating in the SCC and the genes in the rest of the network were compared. Median betweenness centrality in the SCC and global networks were 123 and 0, respectively; the distributions in the two groups differed significantly (Mann–Whitney Wilcoxon *W* = 163, *n*1 = 16, *n*2 = 90, *p-value* = 1.406e-10) supporting the central role of the regulatory core in the global network. It should be noted that the betweenness centrality is more sensitive than other topological features such as degree or clustering coefficient to data incompleteness (missing genes or interactions) because it depends on the global network structure [13,14].

Having the hubs identified, we asked the question whether the strong connectivity occurs between genes involved in common or distinct biological processes. Modules (clusters of genes sharing functional or topological properties) in the network were distinguished by assigning the pathological prion disease processes (derived from gene ontology annotations, described by Hwang *et al.*) to genes constituting the network core. Four modules were considered: disease-causing prion protein (PrP^Sc^) replication and accumulation, immune response, neuronal cell death and other functions (genes which could not be assigned to any of the previous groups). Inter-modular participation is a measure for identifying genes which link different biological processes and this measure was calculated for all module members. Three groups can be distinguished according to node role (see materials and methods): (1) one connector hub with high inter-modular participation (P > 0.60) and significant within-module connectivity (z >2.5) at the same time highly central (normalized centrality >1): *TGFB1*; (2) satellite connectors, (genes with weak connectivity to other nodes of same function but with high ratio of connections to other modules) that share high centrality (normalized centrality >1): CSF1, TLR2, LGALS3 and STAT3; (3) less high central satellite connectors (positive normalized centrality): CEBPD, STAT1 and B2M; (4) other non-central but inter-module participative genes that are regulated by the SCC and are associated with a different functional category than the regulated gene or are regulating genes of other functions: CASP1, CLU, TGFBR2, P2RX7, NFATC1, CXCL10, CCND1, CYBB, AIF1 and GFAP. As expected most of the selected hubs and connectors are parts of SCC supporting its assumed role as a transition driver.

### Functional analysis

**Figure 3:**
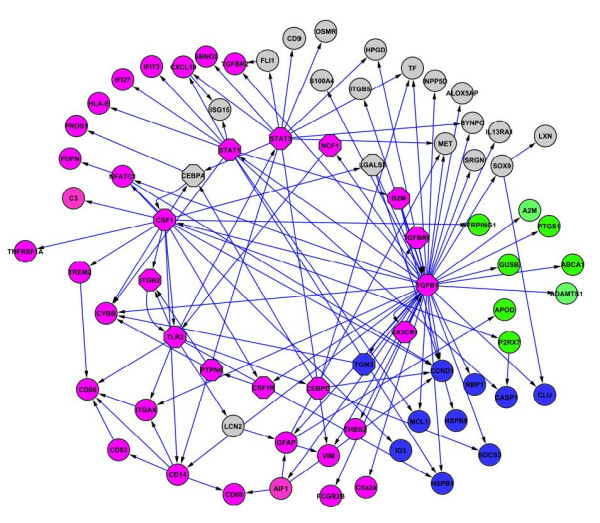
**Functional analysis of core network with pathological features Genes associated with PrP**^**Sc**^**replication and accumulation are in green, with nerve cell death in blue, with immune response (including, microglia/astrocyte activation, leukocyte extravasation, general immune response) in pink.** Other genes are indicated in grey. SCC genes are indicated as octagons.

We have categorized the genes of the core network with regard to the four pathological features described by Hwang *et al.* (Figure 3, Table 1). No genes from the pathological feature category synaptic degeneration were found in the core network, but it should be noted that only one of the 333 DEGs in the original mouse study was a member of this category.

**Table 1:** Summary of the genes and their functional categories

**Biological function**		**Genes**^**a**^
**Prp(Sc replication and accumulation**		A2M, ABCA1, ADAMTS1, APOD, PTGS1, SERPING1
**Immune response**		
	Complement activation: complement system	C3
	Complement activation: coagulation & kallikrein system	PDPN, PROS1
	Pattern recognition and other receptor	CD14, CD68, **ITGB2**, FCGR2B, TREM2, **TLR2**
	Microglia/astrocyte activation related	GFAP, **PTPN6**, **STAT1**, **STAT3**, THBS2, TNFRSF1A, VIM
	Cytokine, chemokine and growth factor related	**CSF1**, **CSF1R**, CXCL10, **CX3CR**1
	Leukocyte extravasation	CYBB, ITGAX, **NCF1**, **TGFB1**, TGFBR1, TGFBR2
	Other immune response	AIF1, **B2M**, CD83, CD86, **CEBPA**, **CEBPD**, Ctla2a, HLA-E, IFI27, IFIT3, NFATC1, SBNO2
**Cell death**		CASP1, CCN D1, CLU, HSPB1, HSPB8, ID3, MCL1, RBP1, SOCS3, **TGM2**
**Other**		ALOX5AP, CD9, FLI1, GUSB, HPGD, IL13RA1, INPP5D, ISG15, LCN2, **LGAL53**, LXN, MET, OSMR, P2RX7, S100A4, SOX9, SRGN, SYNPO, TF

^a^Genes in the SCC are indicated in bold.

A potential sequence of reactive changes has been proposed by Hwang *et al.* and we have been able to identify genes in our SCC and core network at each stage in this proposed sequence. Transcriptome analysis suggested that one of the first changes was the activation of the complement pathways: the complement factor *C3* is located in the core network. Also, pattern recognition receptors (PRRs) and other receptors may potentially recognize PrP^Sc^: *ITGB2* and *TLR2* in the SCC; *CD14*, *CD68*, *FCGR2B*, *TREM2* in the core network. Subsequently, the complement complexes and PRRs may be responsible for stimulating the production of cytokines (*CSF1* in the SCC and *CXCL10* in the core network) and growth factors (*TGFB1* in the SCC). They may also activate astrocytes, indicated by the increased expression of the glial marker *GFAP* in the core network. Cytokines released by microglia and astrocytes then lead to the activation of endothelial cells, which would stimulate the migration of leukocytes from the blood, followed by their differentiation into microglia (leukocyte extravasation), involving *ITGB2*, *NCF1*, *TGFBR1*, *TGFB1* in the SCC and *TGFBR2*, *ITGAX*, *CYBB* in the core. The upregulation of CSF1 (in the SCC) suggests that mononuclear leukocytes (blood monocytes) may be converted into microglia upon entering the brain.

### Robustness of the results

In order to assess the effect the stringency in gene selection criteria could have on the network topology and dynamics, we reconstructed two additional networks based on either more relaxed or constraint criteria for the gene selection. This resulted, as expected, in a bigger and smaller network respectively (see Additional file 1).

From the original list of 333 DEGs we selected genes with p-value smaller than 0.01 on at least one time point among three last time points and obtained a list of 226 differentially expressed genes resulting in a network of 27 nodes and 39 edges. Within this network we identified a SCC of 8 nodes and 13 edges (a subset of the original SCC with 16 nodes and 28 edges). This network again showed a key role of genes involved in the inflammatory response and identical dynamical behavior with two stable states matching experimental expression values in healthy and diseased states. The betweenness centrality of this network is significantly different between genes participating in the SCC and genes in the rest of the network. Median betweenness centrality in the SCC and the reduced version of the global networks were 52.8 and 0, respectively; the distributions in the two groups differed significantly (Mann–Whitney Wilcoxon *W* = 3, *n*1 = 8, *n*2 = 19, *p-value* = 2.358e-5) supporting the central role of the new regulatory core in the reduced version of the global network.

The fragmentation analysis of this reduced version of the global network produced the following results. The mean of the giant component size for 1000 randomized removals of 16 nodes was 13.88 nodes (standard deviation 3.41), while it was only 5.00 nodes in the case of the removal of the 9 remaining nodes from the original 16 (seven genes from the original 16 belonging to the SCC are not included in this smaller network). The difference between these values is 2.59 times the standard deviation of the random removal values. This indicates that despite the missing information it is still possible to detect the importance in terms of connectivity of the 9 remaining key genes belonging to the SCC.

Next, we selected additional genes to expand the original network. This time we queried Prion Protein Data Base ( http://www.prion.systemsbiology.net) to include genes previously not regarded as differentially expressed. To limit the size of this extension we applied three step filtering: 1) we looked for genes that could be upstream and downstream of the original network (the same Pathway Studio settings as in constructing original network); 2) p-value less than 0.01 among three last time points of at least one prion-mouse strain combination; 3) sum of p-values from all time points and prion-mouse combinations less than mean. Applying this criteria we obtained a list of 62 additional genes resulting in a network of 119 nodes and 389 edges. Within this network we obtained one SCC of 82 nodes and 320 edges in which the original SCC of 16 genes were embedded. This network showed again a key role of genes involved in the inflammatory response and identical dynamical behavior with two stable states matching experimental expression values in healthy and diseased states. The betweenness centrality is again significantly different between genes participating in the SCC and genes in the rest of the network. Median betweenness centrality in the SCC and the extended version of the global networks were 92.4 and 0, respectively; the distributions in the two groups differed significantly (Mann–Whitney Wilcoxon *W* = 326, *n*1 = 83, *n*2 = 35, *p-value* = 1.02e-11). However, no significant differences could be found between the betweenness centrality of genes related with immune response and the rest of the genes within the SCC in the expanded network proving lack of topological bias of this group of genes.

The fragmentation analysis of this expanded version of the global network produced the following results. The mean of the giant component size for 1000 randomized removals of 16 nodes was 99.58 nodes (standard deviation 2.76), while it was only 87 nodes in the case of the removal of the 16 genes belonging to the original SCC. The difference between these values is 4.54 times the standard deviation of the random removal values. This indicates that despite the noisy information the 16 nodes belonging to the original SCC still show a remarkable connectivity importance.

In summary, the analysis of these reduced and expanded networks supports the robustness of our findings against both noisy and incomplete information. Despite the lack of detailed mechanistic information about specific pathways we still can gain insights into the importance of neuroinflammation and immune response in prion disease and their impact in the network dynamics and disease progression.

## Discussion

Prion diseases are a protein-based infectious diseases of the nervous system that result in progressive and extensive neurodegeneration throughout the brain. The mechanisms leading to neurodegeneration are incompletely understood, but loss of function of Prion^C^, gain-of-toxic function of Prion^Sc^, activation of cellular and in particular ER stress, neuronal death pathways such as autophagy and apoptosis, and chronic brain inflammation all appear to play a role. Experimental *in vitro* or *in vivo* approaches typically investigate one of the potential pathogenic processes at a time, and often quite elegantly demonstrate their relevance in the disease process. However, to investigate which of these processes is the most pathogenic and contributes the most to the disease process, conventional experimental approaches are more limited. To address this issue, we therefore used a network analysis approach and looked for a set of genes determining the stability of the network using a Boolean dynamical system. By identifying a set of core regulatory genes, most of which are part of the immune response, that can lock a larger gene network into a stable diseased state, we found that it is the process of brain inflammation that is the most likely to play the major pathogenic role in prion disease.

Despite the fact that six of the 333 DEGs described by Hwang *et al.* are related to the oxidative stress response, only one of them is included in our global network (*PRDX6*) and none in the core regulatory network. In neurons, the accumulation of misfolded proteins also leads to endoplasmic reticulum (ER) stress. The normal unfolded protein response (UPR), which is a consequence of the ER stress, should function to clear the misfolded proteins. But it is thought that pro-inflammatory cytokines released by microglia lead to an upregulation of ER stress and an atypical form of UPR in neurons [15-17]. Furthermore, it has been hypothesized that prolonged ER stress activates cell death pathways [17,18]. Cellular stress is one of the possible mechanisms triggering neurodegeneration. Necrosis in acute brain injury is a result of the release of nitric oxide, reactive oxygen species, calcium and glutamate [6]. The role of prion in triggering the stress response is still not understood, but a toxic effect caused by free radicals produced due to an imbalance in the intracellular cupper levels has been previously associated with prion disease and other neurodegenerative diseases [19,20]. Whether this imbalance is due to the loss of function (lack of prion directly or indirectly involved in dismutase activity) or the gain of new functions (aggregation of PrP^Sc^) is still unclear, but there is evidence that the toxicity results from prion dependent processes (alteration of PrP-mediated signaling, PrP mislocalization and oligomerization) in murine models of scrapie [21].

The Ccaat-enhance binding protein (CEBP) transcription factor family has been implicated in the differentiation of myelomonocytic cells and the regulation of gene expression during the activation of macrophages [22]. The isoform CEBPA regulates the expression of antioxidant proteins. *CEBPA-deficient* mice were found to be susceptible to hyperoxic conditions and prion was one of the DEGs identified, thus suggesting a role of wild type prion in lung injury [23]. Overexpression of the *CEBPD* isoform is observed in Alzheimer’s [24] and in prion disease [25]. Colony-stimulating factor 1 (CSF1) and its receptor CSF1R are involved in the process of macrophage DNA synthesis, which is induced by the presence of amyloid beta and prion protein. The presence of misfolded amyloid beta precursor and prion protein promotes macrophage survival in bone marrow cells [26]. Integrin beta 2 is encoded by *ITGB2* and is known to selectively bind to neuronal cells damaged by cleaved prion. It is also involved in neuronal cell killing by activated microglial cells [27]. Transglutaminase 2 is encoded by the *TGM2* gene and may contribute to the cross-linking of soluble peptides preventing amyloid formation of alpha-synuclein or prion protein, thus increasing concentrations of soluble toxic oligomers [28]. Overexpression of *TGM2* is also observed in Huntington and Alzheimer’s diseases [29]. *CX3CR1* is part of the complement system and encodes the receptor for the chemokine fractalkine. Antagonists of chemokine receptors have been shown to play a neuroprotective role [30] in several neurodegenerative diseases including prion disease. Also, thiazolopyrimidine derivatives are antagonists of CX3CR1 and have been developed as drugs for prion disease therapy [31]. TGFB1 is a cytokine which is overexpressed in prion disease. Signaling through the TGFB1 receptor is potentially responsible for the chronic inflammatory processes by regulating an atypical microglia phenotype [32]. *LGALS3* encodes galectin-3, a beta-galactoside binding lectin. It has been shown that lectin binding and normal prion (PrP^C^) glycosylation are altered during aging [33]. STAT1 transcription factor signaling is increased in mouse models lacking PrP^C^, indicating a neuroprotective role of PrP^C^[34,35]. The STAT3 transcription factor is important for the anti-apoptotic activity of humanin and therefore for humanin-mediated neuroprotection after Alzheimer disease-related insult [36]. The protective role of humanin was also observed in prion disease [37].

The possible mechanistic links of the remaining genes in the SCC (*TLR2*, *NCF1*, *PTPN6* and *B2M*) to prion disease are still open. Thus, these genes represent a new set with a potential crucial function in prion disease pathogenesis.

The gene product of TLR2, or Toll-like-receptor 2, is a pattern recognition receptor found primarily on innate immunity cells including microglia/macrophages [38,39]). Although toll-like receptors are known to recognize misfolded proteins and trigger stress responses in various neurodegenerative diseases, TLR2 has not been directly linked with the recognition of prion deposition [40]. Interestingly, pharmacological inhibitors of Toll-like-receptors, including TLR2, are being studied for the treatment of various inflammatory diseases [41]). The gene product of NCF1, or Neutrophil-cytosolic-factor 1, is a component of the neutrophil NADPH, and enzyme complex that produces a superoxide anion, a reactive radical [42]. The gene product of PTPN6, also called SHP-1 (Src homology region 2 domain-containing phosphatase-1), codes for Tyrosine-protein-phosphatase non-receptor type 6, a protein tyrosine phosphatase involved in cell differentiation, in particular hematopoetic cells [43]. B2M codes for β_2_ microglobulin, a component of MHC class I molecules, and its expression in neurons is regulated during injury [44]. These factors point again to a major involvement of the innate immune system in the pathogenesis of prion disease, and furthermore suggest the existence of new targets for possible therapeutic intervention.

In another transcriptomics study by Sorensen *et al.*[25], C57BL/6 or VM mice were inoculated intracerebrally with three strains of scrapie prion that differed from those used in the study by Hwang *et al.* Seventeen genes were found to be related to inflammation and microglia activation. Ten of these genes were also among the 333 DEG in the study by Hwang *et al.*: six are located in our core network (*ABCA1*, *CLU*, *TF* and *SOX9*) and two in our SCC (*B2M* and *TGFB1*). However, it should be noted that *CLU* was placed in the cell death category and *TF* into the iron homeostasis category by Hwang *et al.* All genes were similarly upregulated in both studies. We decided to follow up with the network analysis of data from the latter publication since it was based on larger number of mice model -prion strain combinations and therefore provided more comprehensive list of DEGs. Although, both Hwang’s and Sorensen’s studies included analysis aimed on identifying genes related to inflammatory processes, a stability and dynamics analysis of the gene regulatory network was performed for the first time in the present study, allowing for the identification of brain inflammation as the main prion disease process among all the possible others.

Inflammation response could be an overrepresented functional category in a great number of diseases. In the particular case of neurodegenerative diseases belonging to the class of protein misfolding diseases, it is well established that neuroinflammation plays a role. What we consider remarkable and constitutes our main finding is the key role that neuroinflammation plays in the specific case of prion disease, connecting different functional modules and constituting a switch that allows the network to reach a self-maintained disease state, once triggering factors (protein deposition and the formation of amyloid plaques) initiate the process. According to our simulations, the special topology that connects neuroinflammation elements (a cluster of positive feed-back loops or SCC) makes the regulatory core sensitive under perturbation to easily transit from inactive (healthy) to active (diseased) states but become very stable once the active state is reached.

To experimentally test the role of the genes of the identified master regulatory core in prion disease, we envisage inoculating Prion^Sc^ into knockout mice that are lacking one of the 16 SCC genes. Subsequently, the mice would be analyzed pathologically with regard to neuronal function and death, as well as for DEGs for which the network analysis would be repeated. This analysis would be particularly interesting for the factors whose explicit role has never been demonstrated in prion pathogenesis (*TLR2*, *NCF1*, *PTPN6)*.

## Conclusions

In this study, we have carried out a network analysis based on a recent study of prion disease in mouse in order to gain insights into disease progression. We have built a gene regulatory network consisting of genes differentially expressed due to prion disease infection and analyzed network topology and dynamics to identify key network structures in disease progression. We have identified an SCC that plays a crucial regulatory role in the network and is capable of determining network stability. This design resembles the “Medusa model” described by Kauffman [45], in which a set of genes represents a regulatory head and the remaining genes represent arms controlled by the head.

The sixteen genes of SCC we have identified as master regulatory core are capable of activating 58 further genes which are differentially expressed in several mouse prion models. These genes determine the stability of the network and are also the critical connecting element between different pathological processes (disease-causing prion replication and accumulation, immune response and neuronal cell death).

Perturbation analysis of regulatory core genes demonstrates that each gene is individually capable of triggering the transition between two stable states in the larger network. A switch to the “on” state of any of the regulatory core locks the network the new stable, but diseased, state. We hypothesize that this locking may be the cause of the sustained immune response observed in prion disease, which could ultimately lead to prominent neuronal cell death and the manifestation of clinical symptom

## Methods

### Generation of the global and core regulatory networks

The procedure for the network reconstruction consists of the following steps: a) Obtaining a list of differentially expressed genes. b) Connecting these genes using expression regulatory interactions from literature. c) Determining the core regulatory network.

a) Obtaining a list of differentially expressed genes.

A list of 333 DEGs was extracted from the results of gene expression analysis experiments performed by Hwang *et al.*[4]. These DEGs were found in all five prion-wild type mouse combinations in the study.

b) Connecting differentially expressed genes using gene regulatory interactions described in literature.

The ResNet mammalian database from Ariadne Genomics http://www.ariadnegenomics.com/) was used to construct a gene regulatory network. The ResNet database includes biological relationships and associations, which have been extracted from the biomedical literature using Ariadne's MedScan technology [46,47]. MedScan processes sentences from PubMed abstracts and produces a set of regularized logical structures representing the meaning of each sentence. The ResNet mammalian database stores information harvested from the entire PubMed, including over 715,000 relations for 106,139 proteins, 1220 small molecules, 2175 cellular processes and 3930 diseases. The focus of this database is solely on human, mouse and rat.

We used the list of differentially expressed genes to build a gene regulatory network without including any additional genes not found in microarray experiments resulting in a raw connected graph of 125 nodes and 255 interactions of known effect (positive or negative).To build the network we included only literature evidences of gene expresion regulation (directed and signed interactions) and is therefore smaller and sparser than it would have been if all possible known interactions had been included (i.e. undirected protein-protein interactions, indirect interactions). The expression patterns of the DEGs were checked in the Prion Database ( http://prion.systemsbiology.net) to compare with topology and associations’ logic leading to removal of inconsistent 15 nodes and 81 edges. Additionally, discovery of few errors in text mining process lead us to further validation of the network. To avoid false associations we took all sentences used by Pathway Studio (Ariadne Genomics) to determine gene associations and searched for co-occurrence of specific words: modifiers of sentence meaning, indicating increased risk of false interpretation. In the next phase we checked manually highly uncertain sentences and found two clearly wrong associations: CD86 --+ > TGFB1 and CEBPA --+ > CASP8. In summary, we obtained a final graph of 106 nodes and 169 edges we used for fragmentation analysis in this paper. References for both raw and global network interactions are included in the Additional file 1.

c) Determining the core regulatory network.

Given that only genes with incoming interactions are relevant to the stability analysis, we had to identify genes involved in regulatory feed-back loops, or circuits, and genes regulated by them. For the first task we looked for strongly connected components (SCCs) in the raw network using Binom plugin [48] in Cytoscape [49].

An SCC is a network of nodes, where each node can be accessed directly or indirectly from every other node within the network or, in other words, if there exists a path from each node in the network to every other node. Due to the specific connectivity in a SCC, the information can flow from one node to any other in the structure following at least one path. Such a path has to respect the sense of the interactions (otherwise the component is not strongly but weakly connected). Therefore, the state of any node in the SCC can directly or indirectly affect the state of any other node. This mutual influence between any pair of nodes within the SCC indicates that the SCC may be a relevant stability-related structure. We obtained a single SCC with 16 nodes. After that, we expanded these cores iteratively by adding first neighbors regulated by the SCC until no further neighbors could be added. This yielded a network consisting of the SCC and genes that are directly or indirectly regulated by genes in the SCC (we call this the “core network”). The core network including 74 nodes and 125 interactions (all nodes with incoming interactions) was used for stability and centrality analysis in this paper. References for the interactions of the core network are included in the Additional file 1.

### Stability and perturbation analysis

For the stability analysis we used the SQUAD software package [50], creating a discrete dynamical system that allowed us to identify all the stable states of the system with an asynchronous updating scheme [51] using a binary decision diagram based algorithm [52]. Subsequently, a continuous dynamical system was created to identify the stable states in this continuous model which are located near to the stable states of the discrete system, according to the method described by Mendoza *et al.*, 2006 [53], where the stable states of a Boolean model are taken as initial conditions in the continuous model. Gene perturbations were simulated in the continuous model changing the expression values of specific genes. We also calculated the stability of the SCC in isolation, as well as the stability of the core network. More details about stable states computation and perturbation simulations are included in the Additional file 1.

### Network properties

Fragmentation, betweenness centrality and inter-modular participation measurements were employed to compare the properties of SCC genes with other genes in the network, and to determine key genes that might be potential candidates for experimental validation.

In order to test the importance of the SCC in the network’s connectivity, we examined the fragmentation effect of removing the 16 nodes belonging to the SCC in comparison with the fragmentation effect of 1000 different randomized removals of 16 nodes in the global network of 106 nodes. The giant component is the biggest connected subgraph found in the network for the given fragmentation and thus a good measure for evaluating such fragmentation [54,55].

Betweenness centrality was computed for all genes in the network. The higher the value, the more central the gene is in the network of reference, i.e. other genes are more likely to be connected along the pathway involving these genes [54] (see Additional file 1).

Modules in the global network were defined by functional and pathological process annotation of genes. The participation coefficient P is a measure quantifying inter-modular connections of genes. For any gene in question P is greater than 0, if odds of inter-modular degree to total degree of the gene is less than 1, which means it has to have at least one connection within its own and neighboring modules. Together with measure of within-module connectivity, participation allows to define role a node in the network ranging from most influential global hub till peripheral node (global hub, connector hub, provincial hub, kinless node, satellite connector, peripheral node and ultra-peripheral node). Such genes connect various functional pathways and might therefore be considered key regulators of cellular processes [56].

### Functional analysis

Hwang *et al.* described four pathological features, which were derived from GO attributes: (1) PrP^Sc^ replication and accumulation, (2) microglia/astrocyte activation (which we are calling immune response), (3) synaptic degeneration and (4) neuronal cell death. We mapped these pathological features on the nodes in our core network and examined how the genes in our network may relate to disease progression.

## Competing interests

The authors declare that they have no competing interests.

## Authors’ contributions

IC participated in network reconstruction and analysis, the design of the study and drafted the manuscript, KR participated in the network reconstruction and analysis, the design of the study and drafted the manuscript, WJ participated in the network reconstruction and analysis, participated in the design and coordination of the study and drafted the manuscript, HK conceived of the study, AdS conceived of the study, participated in its design and coordination and helped to draft the manuscript. All authors read and approved the final manuscript.

## Supplementary Material

Additional file 1 Supplementary material. Click here for file
